# Opposite Sides of *Pantoea agglomerans* and Its Associated Commercial Outlook

**DOI:** 10.3390/microorganisms10102072

**Published:** 2022-10-20

**Authors:** Adriana Sturion Lorenzi, Maria Letícia Bonatelli, Mathias Ahii Chia, Leonardo Peressim, Maria Carolina Quecine

**Affiliations:** 1 Department of Cellular Biology, Institute of Biological Sciences, University of Brasília, UnB, Brasília 70910-900, DF, Brazil; 2 Department of Environmental Microbiology, Helmholtz Centre for Environmental Research GmbH—UFZ, 04318 Leipzig, Germany; 3 Department of Botany, Ahmadu Bello University, Zaria 810211, Nigeria; 4 Department of Genetics, “Luiz de Queiroz” College of Agriculture, University of São Paulo, USP, Piracicaba 13418-900, SP, Brazil

**Keywords:** bioprospection, bacterial biofertilizer, commercial outlook, biological risks, antimicrobial activity, genomic analysis

## Abstract

Multifaceted microorganisms such as the bacterium *Pantoea* colonize a wide range of habitats and can exhibit both beneficial and harmful behaviors, which provide new insights into microbial ecology. In the agricultural context, several strains of *Pantoea* spp. can promote plant growth through direct or indirect mechanisms. Members of this genus contribute to plant growth mainly by increasing the supply of nitrogen, solubilizing ammonia and inorganic phosphate, and producing phytohormones (e.g., auxins). Several other studies have shown the potential of strains of *Pantoea* spp. to induce systemic resistance and protection against pests and pathogenic microorganisms in cultivated plants. Strains of the species *Pantoea agglomerans* deserve attention as a pest and phytopathogen control agent. Several of them also possess a biotechnological potential for therapeutic purposes (e.g., immunomodulators) and are implicated in human infections. Thus, the differentiation between the harmful and beneficial strains of *P. agglomerans* is mandatory to apply this bacterium safely as a biofertilizer or biocontroller. This review specifically evaluates the potential of the strain-associated features of *P. agglomerans* for bioprospecting and agricultural applications through its biological versatility as well as clarifying its potential animal and human health risks from a genomic point of view.

## 1. Introduction

The genus *Pantoea* comprises a very diverse group of bacteria, described first by Gavini et al. [1]. This genus belongs to the Enterobacteriaceae family and currently includes 19 species: *Pantoea eucalyptii*, *Pantoea agglomerans*, *Pantoea vagans*, *Pantoea conspicua*, *Pantoea deleyi*, *Pantoea anthophila*, *Pantoea brenneri*, *Pantoea ananatis*, *Pantoea allii*, *Pantoea stewartii*, *Pantoea cypripedii*, *Pantoea calida*, *Pantoea gavinae*, *Pantoea dispersa*, *Pantoea séptica*, *Pantoea wallisii*, *Pantoea eucrina*, *Pantoea rodasii,* and *Pantoea rwandensis* [2,3]. *Pantoea* consists of a cosmopolitan, Gram-negative, and non-encapsulated genus with no sporulation capacity and has been isolated from different habitats, including plant surfaces, buckwheat seeds, and human feces [2]. 

**Figure 1:**
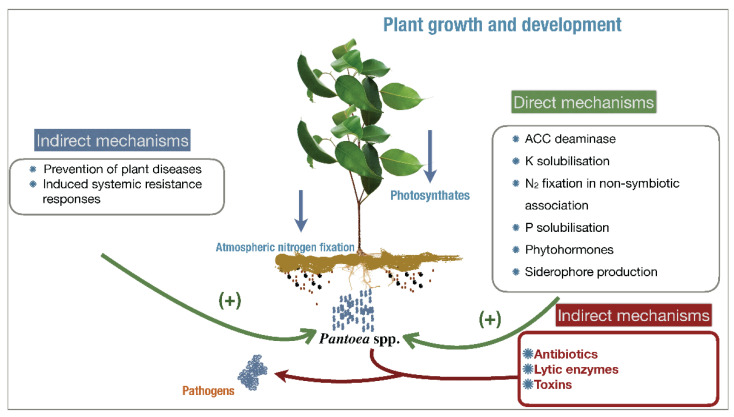
Direct and indirect mechanisms for plant growth by *Pantoea agglomerans*. ACC: 1-aminocyclopropane-1-carboxylate deaminase.

Several strains of *Pantoea* spp. are considered to be plant growth-promoting bacteria (PGPB) through direct and indirect mechanisms (Figure 1). The direct mechanisms employ bacterial traits that result in plant growth promotion directly, including the production of phytohormones, nitrogen fixation, ammonia and phosphorous solubilization, and the sequestration of iron by bacterial siderophores [4,5,6]. The indirect mechanisms comprise bacterial traits that inhibit both plant pathogenic organisms—fungi and bacteria—by the production of antibiotics, cell wall-degrading enzymes, siderophores, competition, hydrogen cyanide, and induced systemic resistance [6,7]. These characteristics are indicative of a great versatility for agricultural applications. 

The bioprospecting of strains of *Pantoea* spp. has yielded commercial antimicrobial agents such as BlightBan C9-1 and Bloomtime Biological for the biocontrol of fire blight, a bacterial disease that affects apple and pear trees [8]. Moreover, a few strains of *Pantoea* spp. can produce immunomodulators, which are substances that increase the immune response against melanomas, infections, allergies, and reverse immunosuppression [9,10,11]. Recent findings have reported potent antiplasmodial alkaloids from an isolate of the genus as hemozoin modulators [12]. In addition, other strains are great bioremediation agents, with the ability to break down herbicides without generating toxic by-products [13].

Despite their outstanding biotechnological potential, several representatives of *Pantoea* are recognized as plant pathogens, causing galls, wilt, rot, and necrosis in a range of relevant plants [8]. Other members of this genus are associated with a wide variety of hosts, including insects, humans, and other animals [14,15,16,17,18]. Even though *Pantoea* is mostly considered to be a plant pathogen, it remains uncertain if it is a deadly pathogen, harmless commensal, or versatile opportunist in humans. *Pantoea* spp. has been reported in wounds, fractures, surface swabs, sputum, urine, ear swabs, oropharynx swabs, knee lacerations, abscesses, dialysates, and blood, among others [18,19,20]. A review with possible associated human diseases/conditions can be found in Walterson and Stavrinides [8]. 

The cosmopolitan distribution, genetic diversity, and metabolic versatility of the strains of *Pantoea* spp. highlight their potential for the development of medical and agricultural products of commercial importance. Nevertheless, there is still a need to better understand their potential harmful effects. This review aims to evaluate the strain-associated features of *Pantoea agglomerans* from a genomic point of view, focusing on how genomics can be used for biotechnological and agricultural applications and what risks it might reveal to animal and human health. 

## 2. Bioprospecting of *P. agglomerans* and Its Commercial Outlook

In Germany’s official regulation TRBA 466 ‘Classification of prokaryotes (bacteria and archaea) into risk groups’ [21], several bacteria found in environmental and host-associated microbiomes belong to species in risk group 2. This is the case of *P. agglomerans*, in which the probiotic or pathogenic trait is a strain-associated feature and not a species-specific trait [21]. 

*P. agglomerans* LMG 1286, which was isolated from a human knee laceration, is the type strain of *P. agglomerans* (previously known as *Enterobacter agglomerans* and *Erwinia herbicola*) [1]. Many *P. agglomerans* isolates possess interesting characteristics for bioprospecting, including potential benefits in agriculture as a pest and phytopathogen control agent [22,23,24], plant growth promoter [5,25], and a superb source of enzymes and other proteins required by plants [26].

When associated with plants, *P. agglomerans* is found in the rhizosphere, on plant surfaces as an epiphyte, and inside plants as an endophyte [6,27,28,29]. It also occurs abundantly in soil and water [8], dust and air [30], bodies of arthropods [31], and is associated with plants and animals, including humans [17]. 

Strains of *P. agglomerans* associated with the rhizosphere and plant tissues efficiently promote the growth of many cultivated plants such as the staple foods rice [32,33] and wheat [34,35], mostly by a combination of different plant growth-promoting mechanisms. Among these combined strain-associated mechanisms to promote plant growth are the atmospheric nitrogen-fixing capacity, production of phytohormones, phytate degradation, phosphate solubilization (i.e., making soil phosphorus bioavailable), ammonia solubilization, antagonistic effects against many plant pathogens (e.g., bacteria and fungi) via the production of antibiotics, competition mechanisms, and resistance induction in plants. Consequently, strains of *P. agglomerans* are recognized as great candidates for an environmentally friendly agriculture, reducing the excessive use of chemical fertilizers and pesticides in the environment. The biological activities of *P. agglomerans* strains that prevent and, in several cases, treat human and animal diseases as well as undertake the bioremediation of polluted environments deeply increase their commercial outlook. Here, we have revised a few single or combined traits of *P. agglomerans* strains with practical commercial applications in different contexts.

### 2.1. Agricultural Outlook of P. agglomerans as a Biofertilizer 

#### 2.1.1. Potassium and Phosphate Solubilization

Although potassium (K) compounds are well-solubilized in soil, native potassium-solubilizing bacteria play an important role as a biofertilizer for the reduction of K chemical fertilizer consumption in cultivated plants. This element is an essential macronutrient for increasing rice growth and grain yield; it is absorbed by rice plants in a greater amount compared with nitrogen (N) and phosphorus (P) [36,37]. K fertilizer compounds such as potassium sulfate (K_2_SO_4_) and potassium chloride (KCl) are usually used in a large amount to overcome a deficiency of K in rice production [38]. In experiments performed by Yaghoubi et al. [39] in both pot and field conditions, *P. agglomerans*, among other potassium-solubilizing bacteria isolated from paddy fields, significantly increased the grain yield (GY) by 20–38% in the pot and 20–52% in the field compared with the control, especially when half of the recommended potassium fertilizer was applied (K_2_SO_4_, 44% K_2_O) to the rice cultivation. Therefore, inoculants containing this bacterium represent a powerful technology for a half reduction of K chemical fertilizer consumption in rice production systems.

On the other hand, phosphorus (P) is a limiting macronutrient because of its low bioavailability in soil. Isolates of *P. agglomerans* have been reported in several contexts as a potential biofertilizer that enhances the release of phosphorus from insoluble compounds in soil for a more sustainable agriculture. Saadouli et al. [40] evaluated the impact of the inoculation of a phosphate-solubilizing *P. agglomerans* strain V8R67 on the phosphorus availability and bacterial community dynamics of a semi-arid soil. The in vitro solubilization of Ca_3_ (PO_4_)_2_ by V8R67 was highly efficient (980 mg/L) and was associated with a drop in pH due to the secretion of gluconic acid. These effects were concomitant with the detection of *gdh* and *pqq*C genes involved in the expression of the MPS (mineral phosphate solubilization) phenotype in *P. agglomerans* V8R67. Additionally, the content of available P in the semi-arid soil significantly increased by 69%. As stated by the authors, this improvement in the phosphate availability suggested that V8R67 represents a promising environmentally friendly biofertilizer in arid and semi-arid environments.

The phosphate-solubilizing *P. agglomerans* strain ZB isolated from the rhizosphere soil of Araucaria showed a great ability to solubilize different insoluble inorganic phosphate sources through Ca_3_(PO_4_)_2_ (TCP), hydroxyapatite (HP), CaHPO_4_, AlPO_4_, FePO_4_, and rock phosphates (RPs). A positive correlation between the production of organic acids and phosphate solubilization was found. Moreover, *P. agglomerans* ZB possesses many plant growth promotion traits such as the production of IAA (indole 3-acetic acid), phytase, alkaline phosphatase, and a N_2_ fixation capacity [41].

Although liquid formulations have a longer shelf life, *P. agglomerans* strain P5 was used in solid formulations of phosphate-solubilizing bioinoculants in Iran [42]. The same strain (P5) was studied for the effect of room temperature storage on the initial number of viable cells (2 × 10^8^ CFU mL^−1^, CFU—colony forming unit) in liquid formulations. After three months, the bacterial population found was 10^6^ CFU mL^−1^, which clearly showed a decrease in the number of viable cells over the course of time [43]. 

#### 2.1.2. Nitrogen Fixation, IAA, and Siderophore Production 

Cell viability is a measure of the proportion of healthy cells within a population to ensure its survival. Thus, the number of bacteria applied to seeds is of fundamental importance to achieve the effectiveness of inoculants [44,45]. Quality standards for inoculants vary among countries, but the number of bacteria required for the optimum performance of inoculants mostly ranges from 5 × 10^7^ to 1 × 10^9^ CFU g^−1^ or mL^−1^ [46]. Considerations must be given to the minimum number of viable cells per seed after the application of an inoculant at the rate recommended by the manufacturers to secure high efficiency. The minimum amounts of rhizobacteria usually accepted per seed are 10^3^ for small seeds, 10^4^ for medium seeds, and 10^5^ for large seeds such as soybeans [46,47].

In this context, an interesting approach was used with *P. agglomerans* strain ISIB55, which was isolated from a soybean. ISIB55 was immobilized in nanofibers and evaluated for the stability of its potentially beneficial characteristics for plant growth promotion (nitrogen fixation, the production of IAA, phosphate solubilization, and the synthesis of siderophores) on soybean plants [48]. The study also evaluated the effect of coating the soybean seeds with immobilized bacteria on bacterial survival during seed storage as well as the germination and growth parameters of the plant. This approach was successful as the nanofibers immobilized with *P. agglomerans* ISIB55 maintained the viability and beneficial properties of the rhizobacterium, which upheld the ability to fix nitrogen, produce IAA, solubilize phosphate, and synthesize siderophores after 30 days of seed storage. The number of viable cells was approximately 10^5^ CFU of *P. agglomerans* ISIB55 in the seeds of two soybean varieties, TEC 5936 IPRO and BRASMAX PONTA IPRO (RSF 7166 IPRO), coated with immobilized rhizobacterium in nanofibers with an initial cell concentration of 10^9^ CFU g^−1^. A seed coating with *P. agglomerans* ISIB55 increased the germination, length, and dry weight of the root. The authors concluded that the technology employed in the study could be considered to be an ecologically promising approach to maximize soybean production through a bacterial inoculant [48].

### 2.2. Agricultural Outlook of P. agglomerans as a Biocontroller

In the context of biocontrol, *P. agglomerans* strain CPA-2, isolated from a fruit surface, was effective in controlling postharvest pathogens in citrus and pome fruit [49]. CPA-2 biocontrols *Penicillium digitatum* and *P. italicum* in oranges. Several studies have provided evidence of a potentially cost-effective way of the large-scale industrial production of *P. agglomerans*. In this sense, Costa et al. [50] evaluated different sources of nitrogen and carbon to maximize the production of the biomass of CPA-2 at a low cost, maintaining the effectiveness of the biocontrol in commercial products and by-products. *P. agglomerans* could be cultured using a combination of nitrogen sources, including yeast extract (5 g L^−1^) and dry beer yeast (10 g L^−1^), with low-cost carbohydrates such as sucrose (10 g L^−1^) and molasses (20 g L^−1^), respectively. The use of preparations containing the bacterium also efficiently protected citrus fruit against mold. An interesting review of food preservation can be found in Dutkiewicz et al. [51].

A study by Haidar et al. [52] showed that *P. agglomerans* S1 was active in biocontrolling *Neofusicoccum parvum* in a grapevine through the combination of its antifungal activity and its ability to activate the grapevine defense system. A co-culture of the durum wheat plant root-associated bacterium *P. agglomerans* Pa and the date palm leaf-derived fungus *Penicillium citrinum* was effective in inducing bacterial siderophores with antifungal effects against both *P. citrinum* TDPEF34 and *Aspergillus niger* IMI51433 [7]. Moreover, the Pa strain induced a stress tolerance in durum wheat, with interesting salt-stress alleviation and plant growth-promoting activities (PGPAs) that included hormone auxin biosynthesis, the production of 1-aminocyclopropane-1-carboxylate (ACC) deaminase, and ammonia and phosphate solubilization [6]. 

Notably, strains of *P. agglomerans* are capable of producing antibiotics that differ among isolates and have a diverse scope of targets to fight plant, animal, and human pathogens as well as to preserve food. Several compounds can usually be produced by a single strain, which allows an antimicrobial effect achieved by synergism [13]. A cluster of 16 *ehp* plasmid genes is required for the production of the broad-spectrum phenazine antibiotic D-alanylgriseoluteic acid (AGA) by *P. agglomerans* Eh1087, which has the potential to control fire blight caused by the phytopathogen *Erwinia amylovora* [53]. Pantocin A (also known as herbicolin O) and pantocin B are peptide-based antibiotics produced by *P. agglomerans* Eh318 that biocontrol Gram-negative bacteria, especially the plant pathogen *E. amylovora* (IC50 = 200 nM for pantocin A and IC50 = 750 for pantocin B) [13]. The pantocin A gene cluster consists of a small gene (*paa*P) that encodes a thirty-amino acid precursor peptide and three genes (*paa*ABC) that encode the enzymes necessary to convert this precursor into pantocin A [54]. The pantocin B gene cluster contains 17.5 Kb of DNA that encode for 13 open reading frames (ORFs) (*pab*AM) [54]. Both antibiotics pantocin A and pantocin B inhibit enzymes in the pathways for histidine and arginine biosynthesis in *E. amylovora*, respectively. A molecular analysis of the microcin gene cluster, a peptide antibiotic produced by *P. agglomerans* Eh252, revealed three ORFs (*mpa*A–C) with a high similarity (97 to 99%) to the *paa*AC genes of the pantocin A biosynthesis. In addition, DNA sequences similar to those of the *paa*P gene of pantocin A were also found in Eh252 [55]. According to Jin et al. [56], a 21-gene cluster (*adm*) is associated with the biosynthesis of the andrimid of *P. agglomerans* Eh335, a potent broad-spectrum antibiotic with a high selectivity for prokaryotic acetyl-CoA carboxylase (ACC). Other antibiotics reported from *P. agglomerans* strains are herbicolin A and B [57,58] and agglomerins A, B, C, and D [59]. 

#### Genetic Engineering of an Isolate of *P. agglomerans* for Biocontrol

Many reports describe the endophytic colonization of sugarcane plants by *P. agglomerans* [27,60,61,62,63]. Of particular interest, *P. agglomerans* strain 33.1—endophytically isolated from *Eucalyptus grandis*—has been described as an agent of cross-colonization for sugarcane capable of substantially promoting sugarcane growth [5]. The endophytic strains of *P. agglomerans* are also strong candidates for a transformation with *cry* genes. Among the members of the Cry protein family, Cry1 and Cry2 are of particular interest in agriculture because of their high toxicity to insect larvae of the orders Lepidoptera and Coleoptera as well as nematodes and mites [64,65,66,67]. The transfer of the cry1Ac7 gene to *P. agglomerans* 33.1 resulted in a greater control of the sugarcane borer [25].

### 2.3. Commercial Outlook of P. agglomerans for Human and Environmental Health

A low molecular weight lipopolysaccharide (LPS), identified as the *Pantoea agglomerans* 1 (IP-PA1) immunopotentiator, has a wide variety of therapeutic properties such as the activation of macrophages and dendritic cells (DCs) via toll-like receptor (TLR)-4, a specific receptor of the LPS [10]. IP-PA1 is effective in the prevention and treatment of human and animal disorders such as tumors, hyperlipidemia, diabetes, ulcers, several infectious diseases, atopic allergies, and stress-induced immunosuppression, and possess a strong analgesic effect [10,51]. 

As a symbiont of many insect species, *P. agglomerans* has application in paratransgenesis—the process of genetically transforming the symbiont of an organism to confer a specific function(s) that reduce vector competence to pathogens. Mosquitoes of the genus *Plasmodium* that are vectors of the malaria-causing parasitic protozoan were successfully controlled by paratransgenesis using genetically engineered *P. agglomerans* to express and secrete antiparasitic molecules inside the host [68,69]. Thus, paratransgenesis presents an excellent alternative for the eradication of malaria, a disease that kills more than a million people annually, as well as other diseases spread by insects to humans, animals, and plants.

Recently, Thissera et al. [12] reported potent antiplasmodial alkaloids produced by the rhizobacterium *P. agglomerans* Pa as natural hemozoin modulators with a strong potential to be developed as antimalarial medicaments. Hemozoin formation, an aggregate of hematin (oxidized heme) produced upon hemoglobin digestion by hematophagous organisms, is essential for the survival of these parasites [70]. Therefore, the inhibition of hemozoin formation represents an attractive antimalarial drug target. 

In addition, the genome mining of *P. agglomerans* strains has increasingly shown a large repertoire of genes contributing to beneficial functions, along with gene encoding for secondary metabolites harbored by the core genome and accessory genome, respectively. Smith et al. [71] published a draft genome sequence of the cystic fibrosis *P. agglomerans* Tx10, which produced an effective antibiotic against *Staphylococcus aureus*. In addition to providing the means for identifying antibiotic biosynthetic clusters, the genome mining of Tx10 also allowed for the evaluation of the contribution of natural products to polymicrobial infections in cystic fibrosis.

Finally, *P. agglomerans* strains have also demonstrated potential for the biodegradation activity of various chemical pollutants in soil and water, including petroleum hydrocarbons and toxic metals, as well as the production of hydrogen from waste [72]. Therefore, strains with these associated traits play a valuable bioremediation role that, in several cases, represents an alternative form of energy. Furthermore, biofilms formed by *P. agglomerans* prevent the percolation of industrial contaminants such as zinc oxide nanoparticles in soils [73]. 

## 3. Pathogenic Potential of *P. agglomerans* and Its Associated Biological Risks

### 3.1. Parasitic Colonization of Plants

Pathogenic strains of *P. agglomerans* can cause disease in a wide range of plants, including sweetcorn [74], pea [75], sweet potato [76], sugarcane [77], bamboo [78], wheat [79], rice [80], cotton [74], beet [81], eucalyptus [82,83], and the ornamental plant *Alocasia cucullata* (popular Chinese taro) [84]. 

In table beet, wisteria, the ornamental *Gypsophila paniculate*, and blackberries, the pathogenic strains are tumorogenic, inducing gall formations [81,85,86] that are abnormal outgrowths of plant tissues. A few of these strains have evolved into a hypersensitive response and pathogenicity (*hrp*)-dependent and host-specific gall-forming pathogen by the acquisition of a pathogenicity plasmid containing a type III secretion system (T3SS) and its effectors (T3Es) [87]. Using draft genome sequences and a machine learning approach, Nissan et al. [87] performed an inventory of type III effectors in two *P. agglomerans* gall-forming pathovars (*Pag* strain 824-1 and *Pab* strain 4188). A pathovar is recognized as a bacterial strain or set of strains with the same or similar characteristics that are differentiated at an infrasubspecific level from other strains of the same species or subspecies based on a distinctive pathogenicity to one or more plant hosts [88]. *P. agglomerans* pv. *gypsophilae* (*Pag*), for instance, incites galls on gypsophila (*Gypsophila paniculata*) and a hypersensitive response (HR) in beet (*Beta vulgaris*) whereas *P. agglomerans* pv. *betae* (*Pab*) causes galls on both beet and gypsophila [87]. 

Despite these implications, the diversity of strains—as well as their association with hosts and diseases—remains poorly understood [89], which makes the comprehension of plant–pathogen interactions to prevent plant diseases challenging. 

### 3.2. Host Association with Humans and Animals

Infections caused by *P. agglomerans* strains usually occur accidentally after a perforation or laceration of the skin with plant spines, wood chips, or other plant material containing the bacterium [89]. These accidents are common during agricultural and gardening work and when children play. Septic arthritis or synovitis appear as common clinical symptoms of an exogenous *P. agglomerans* infection. Endophthalmitis, peritonitis, periostitis, endocarditis, and osteomyelitis are among the other known symptoms [86]. Moreover, *P. agglomerans* has been identified as a prevalent bacterium in cotton cultivated worldwide, rarely as a pathogen, but generally as an epiphyte. It is particularly numerous in cotton bracts after the senescence of the plant [30]. During the processing of cotton in factories, the bacterium and its bioproducts are released with the cotton powder into the air and inhaled by workers, causing a respiratory disorder defined as byssinosis [29]. The inhaled substance is an endotoxin, mainly consisting of lipopolysaccharide (LPS), in addition to phospholipids and proteins. Among the pathological effects, acute and chronic inflammation have been reported, resulting in damage to the endothelial cells and the leakage of cells and fluids into the pulmonary interstitium. These changes cause bronchoconstriction, fever, irritation of the airways, and chest tightness [29,90]. Although other microbial and plant constituents are also considered to be potential causative agents of byssinosis (i.e., reactive airway disease), the endotoxin produced by *P. agglomerans* strains and other Gram-negative bacteria in cotton dust is still considered to be a major cause of the disease.

In spite of not being considered a true pathogen in humans, strains of *P. agglomerans* are often found in hospitals [89,91]. Clinical strains of the bacterium are generally opportunistic pathogens, infecting humans through wounds or openings and affecting mainly immunocompromised patients [8]. As an opportunistic human pathogen, this bacterium can occur sporadically or in outbreaks [89]. Infections in hospitalized patients, usually immunodeficient, are mainly associated with medical equipment or fluids contaminated with the bacterium. In the early 1970s, *P. agglomerans* (then called *Enterobacter agglomerans*) was responsible for a major outbreak of septicemia in the USA and Canada involving 25 hospitals. This was caused by contaminated caps on bottles of liquid for infusions, with 378 reported cases [91]. Since this episode, the bacteremia caused by *P. agglomerans* strains has been associated with intravenous fluid contamination, parenteral nutrition, propofol anesthetic agents, blood products, and transfer tubes used for intravenous hydration [89]. Furthermore, *P. agglomerans* have been isolated from joint fluids in patients with arthritis, peritonitis, or osteomyelitis [20].

There is no evidence that *P. agglomerans* clinical strains represent a population distinct from plant pathogenic strains [89]. Compared with humans, there are a few reports of infectious diseases caused by strains of *P. agglomerans* in other vertebrates, which have been linked to abortion, equine placentitis [92,93], a hemorrhagic disease in dolphins (*Coryphaena hippurus*) [94], and allergic lung disease in cattle [95].

## 4. Genomics as a Tool to Explore the Potential and Hazards of *P. agglomerans*

Advances in sequencing technology have made genome sequencing more accessible and affordable. For small genomes, which is the case for most bacteria, achieving complete or near-complete high-quality genomes in a short time is now a reality. Genomic data allow scientists to explore bacteria with beneficial potential applications [96,97], understand their taxonomy [98], and investigate their pathogenic potential [99].

There are several *P. agglomerans* genomes available in public databases. In this review, we focused on the *P. agglomerans* genomes deposited in the National Center for Biotechnology Information (NCBI) database that had a study or data showing their beneficial potential toward plants or the environment. For a comparison, we also retrieved the genomes of *P. agglomerans* capable of causing disease in plants or humans (Table 1). As expected, the genomic data of *P. agglomerans* showed that both the beneficial and harmful strains presented similar metrics. Their genome size ranged from 5.3 to 4.7 Mb, with an average GC percentage of 55%. When all strains were compared, an average of 4530 coding sequences (CDS) were reported (Table 1).

**Figure 2:**
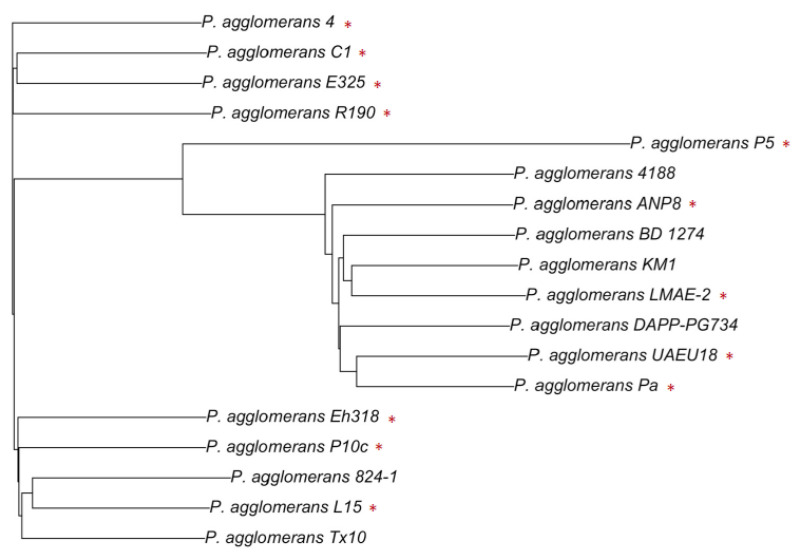
Average nucleotide identity (ANI) distance clustering based on the BIONJ clustering method [132] of *Pantoea agglomerans* strains with beneficial and harmful traits. Red asterisks indicate beneficial strains.

More interestingly, an analysis of the genomic data from the analyzed *P. agglomerans* strains was not able to clearly cluster the beneficial strains (Figure 2). Bacterial relatedness between two strains is often measured by the average nucleotide identity (ANI). To be considered as the same species, two strains should share more than 95% ANI [100], which correlates well with the 70% DNA–DNA reassociation standard of a species definition [99]. The genome-based distance matrix calculator from Rodriguez and Konstantinidis [101] was used to generate the ANI matrix and the cluster based on the ANI.

As expected, *P. agglomerans* strains shared among each other more than 95% ANI (Supplementary Figure S1), but the cluster analysis based on the ANI did not separate the harmful from the beneficial strains (Figure 2). A comprehensive phylogenetic analysis of this species is necessary, but here we show the challenge and complexity of investigating the limits between the harmful and beneficial strains within a species. This was expected, and it has been observed in other species [102,103]. However, genomic data are a valuable resource to investigate genes and the potential of both beneficial and pathogenic strains.

### 4.1. Genomic Potential of the Beneficial Strains of P. agglomerans: From Plant to Environment

The genomes of the *P. agglomerans* beneficial strains contain several genes that could be related to their potential to promote plant growth, to control pathogens, or to bioremediate (Table 1). Here, we highlight the genes and processes reported in their genomes that can be associated with such activities.

Genes related to the direct mechanisms of plant growth promotion such as mineral phosphate solubilization and indole acetic acid (IAA) production [104,105] were found in several beneficial strains. IAA production from tryptophan can happen through different pathways whereas the *ipd*C gene from the indole-3-pyruvate (IPA) pathway and *ami*E and *aam* (homologous to *iaa*M) genes from the indole-3-acetamide (IAM) pathway were found in the strains ANP8 (IPA and IAM), C1 (IPA and IAM), and P5 (IAM) [106,107,108]. In addition, genes involved in the production of gluconic acid, an important organic acid responsible for mineral phosphate solubilization, were found in the beneficial strains. Such genes include glucose dehydrogenase (*gcd*) and those related to pyrroloquinoline quinine synthesis, a coenzyme for *gcd* activity [109], reported in ANP8, C1, and P5 strains [106,107,108]. Interestingly, ANP8 harbors several genes involved in regulating potassium concentrations, which the authors associated with the salinity tolerance potential of this strain [106].

Genes related to indirect plant growth promotion mechanisms (e.g., biocontrol) were also found in the beneficial strains. Volatile organic compounds (VOCs) such as acetoin and 2,3-butanediol can trigger plant systemic resistance and increase their resistance against pathogens [110,111]. Genes associated with the production of VOCs were found in the C1 and P5 genomes as well as genes related to siderophore synthesis, which are high-affinity iron-chelating molecules that can be involved in biocontrol mechanisms [107,108,112,113]. Both strains harbor the genes *ent*ABCEF involved in the synthesis of an enterobactin siderophore [114]. Moreover, genes to produce antibiotics were found in beneficial strains such as pantocin A in the strains P10c and Eh318 and phenazine in the strain R190 [115,116,117].

Finally, genes that might be involved in bioremediation processes were also investigated in the *P. agglomerans* beneficial strains. Bioremediation can be applied to the plant–microbe relationship to promote the bioremediation of contaminated soils [118]. The strain C1 harbors genes related to arsenic, copper, and cadmium tolerance whereas the strain LMAE-2, which was isolated from an environment contaminated with copper, harbors the gene cluster *cop*ABCD that is associated with this metal homeostasis in other bacterial strains [107,119,120,121]. 

### 4.2. Exploring the Hazards of P. agglomerans Strains: What Genomic Data Can Tell Us

The genomes of the pathogenic or potentially pathogenic strains of *P. agglomerans* were accessed and important insights toward their pathogenicity were made. *P. agglomerans* strain KM1 was isolated from kimchi, a Korean vegetable side dish that is prepared through a fermentation process. This strain is thought to be pathogenic based on its genomic proprieties and on its capability of exerting strong immunostimulatory properties on macrophages in vitro [122]. The authors identified virulence factors that were classified into five categories: secretion systems, adhesion, motility, iron uptake/sequestration system, and toxins [122].

The strain KM1 has all the putative genes of the type VI secretion system; the structural gene components hemolysin-co-regulated protein (Hcp) and valine-glycine repeat G (*VgrG*) protein were also validated using PCR and amplicon sequencing [122]. Different types of secretion systems might be related to pathogenicity [123] and the type III secretion system (T3SS) and effectors (T3Es) of *P. agglomerans* strains 824-1 and 4188 are related to their pathogenicity specificity [87]. These two strains are distinct pathovars that provoke different responses in two plants. As previously stated (Section 3), strain 4188 elicits galls on beet and gypsophila whilst 824-1 incites galls on gypsophila and causes a hypersensitive response (HR) in beet. The authors found that the pathogenicity of these strains could be related to the difference in their T3Es [87] whilst the ability of another *P. agglomerans* strain, DAPP-PG 734, to induce an HR in tobacco seemed to be related to the presence of a complete *hrp*/*hrc* gene cluster [124].

In addition, plasmids can play an important role in the bacterial potential to cause disease. The host specificity of the pathogens 4188 and 824-1 is related to the acquisition of a plasmid containing both T3SS and T3Es elements [87]. Moreover, *P. agglomerans* strain BD 1274 possesses a plasmid containing a cluster of genes responsible for the conjugal transfer of DNA; the authors inferred that this played a major role in the pathogenicity of this strain [125].

However, determining the beneficial or hazard potential of a bacterial strain is not trivial. Cherif-Silini et al. [6] searched for genes with putative functions related to plant growth promotion in several *P. agglomerans* strains. They considered genes involved in bacterial colonization, nutrient availability, plant growth hormones, metal tolerance, and more. They were able to find several of these genes in both beneficial and harmful strains. In the same way, Guevarra et al. [122] reported several potential virulence factors in both types of strains; the beneficial and the harmful ones. This was expected at a certain level [126], but showed that, although genome mining is a fundamental step toward understanding the bacterial metabolism and potential, this should be followed by complementary research. In vitro and in vivo assays to evaluate the beneficial or harmful potential of a strain as well as transcriptomic, proteomic and/or metabolomic approaches to analyze the potential of the active metabolic functions should be highly considered. Nevertheless, the interpretation of genomic and transcriptomic data in the context of the biological function is a major challenge due to the influence of specific changes on the phenotypic variation [127]. Thus, a multiomic design provided by the integration of genomics, transcriptomics, proteomics, and/or metabolomics in a specific context enables unprecedented possibilities to elucidate the underlying mechanisms of biological functions and uncover hidden associations among omics.

**Table 1:** Genomic data of *Pantoea agglomerans* strains with beneficial and harmful plant and environmental traits.

Strain	RefSeq	Function	Isolation Source	Location	Genome Size (bp)	GC (%)	Contigs/Scaffolds	Plasmids/Scaffolds of Plasmids	CDS	rRNA	tRNA	tmRNA	Reference
	Beneficial *P. agglomerans* strains												
L15	GCF_003860325.1	Biocontrol	*Hypericum perphoratum* phyllosphere	Poland	4,858,869	55.10	1	3	4455	22	81	1	[128]
UAEU18	GCF_010523255.1	PGPB	Date palm rhizosphere soil	United Arab Emirates	4,825,350	55.20	1	3	4386	22	80	1	[129]
P5	GCF_002157425.2	PGPB	Farmland soil	Iran	5,074,260	54.87	127	-	4684	8	61	1	[108]
C1	GCF_009759885.1	PGPB	Lettuce phyllosphere	Italy	4,846,162	55.16	21	-	4465	12	72	1	[107]
P10c	GCF_001288285.1	Biocontrol	Pear blossoms	New Zealand	4,775,916	55.06	18	2	4381	6	67	1	[115]
ANP8	GCF_017315165.1	PGPB	Alfalfa root nodules	Iran	5,035,017	55.00	1390	-	4404	14	75	1	[106]
Pa	GCF_002082355.1	PGPB	Durum wheat rhizosphere	Algeria	4,795,157	55.02	45	-	4358	13	70	1	[6]
R190	GCF_000731125.1	Biocontrol	Apple orchard	South Korea	5,002,566	55.05	2	3	4592	24	78	1	[117]
LMAE-2	GCF_000814075.1	Bioremediation	Marine sediment	Chile	4,981,165	55.15	155	-	4562	19	80	1	[119]
4	GCF_000743785.2	Biocontrol	Wheat seed	Canada	4,827,890	55.17	4	-	4399	19	74	1	[130]
E325	GCF_014353865.1	Biocontrol	Apple flowers	United States	4,786,783	55.21	162	-	4365	14	74	1	[131]
Eh318	GCF_000687245.1	Biocontrol	Stem from an asymptomatic apple	United States	5,035,839	54.77	34	-	4597	21	74	1	[116]
	Harmful *P. agglomerans* strains												
KM1	GCF_012241415.1	Opportunistic pathogen	Short-term fermented homemade kimchi	South Korea	4,995,756	55.07	34	12	4568	2	64	1	[122]
BD 1274	GCF_003369505.1	Pathogen of onion	Onion seeds	South Africa	4,968,508	54.96	246	-	4612	11	48	1	[125]
824-1	GCF_001661985.1	Gall-forming	Gypsophila	United States	4,989,068	54.81	55	-	4648	7	73	1	[87]
4188	GCF_001662025.1	Gall-forming	Beet	United States	5,001,997	54.96	79	-	4629	10	74	1	[87]
Tx10	GCF_000475055.1	Cystic fibrosis	Sputum of a cystic fibrosis patient	United States	4,856,993	55.10	22	-	4446	16	73	1	[71]
DAPP-PG 734	GCF_000710215.1	Related to olive knot	Olive knots	Italy	5,365,929	54.75	195	-	5000	11	71	1	[124]

CDS: coding sequences.

## 5. Final Considerations

The bioprospecting of *P. agglomerans* enhances its application for biotechnological and agricultural purposes. This may generate high-value products to suppress the development of various plant pathogens and promote the growth of plants of agricultural interest, emerging as a potentially effective biodefensive and/or biofertilizer. Despite the proven pathological effects of this bacterium as a cause of occupational diseases with an allergic and immunotoxic background as well as accidental and opportunistic infections in different environments, the beneficial characteristics of strains belonging to the *P. agglomerans* species are of great interest for a number of applications. Possible restrictions on the use of this bacterium and its bioproducts should be carefully considered, ensuring that precautions are in place to guarantee the maintenance of the health of the environment, animals, and humans. In addition to genome mining, a polyphasic approach that includes other omics-related data is strongly recommended to achieve the potential and realize the hazards of *P. agglomerans* because several important genes can be found in both the beneficial and harmful strains.

## Supplementary Materials

The following supporting information can be downloaded at: https://www.mdpi.com/article/10.3390/microorganisms10102072/s1, Figure S1: Average nucleotide identity (ANI) matrix of *Pantoea agglomerans* strains with beneficial and harmful traits.

## Abbreviations

PGPB: plant growth-promoting bacteria; PGPR: plant growth-promoting rhizobacteria; PGPAs: plant growth-promoting activities; BNF: biological nitrogen fixation; IAA: indole acetic acid; IPS: inorganic phosphate solubilization; LPS: lipopolysaccharide; QS: quorum sensing.
