# Supplementary material for: Opposite Sides of Pantoea agglomerans and Its Associated Commercial Outlook
Source: Microorganisms. 2022 Oct 20;10(10):2072. doi: 10.3390/microorganisms10102072 (PMC9610544; doi:10.3390/microorganisms10102072)
Supplement: Supplementary file 1 [file microorganisms-10-02072-s001.zip › microorganisms-1963339-supplementary.pdf]

	P._agglomerans_LMAE−2	P._agglomerans_DAPP−PG734	P._agglomerans_KM1	P._agglomerans_BD_1274	P._agglomerans_Pa	P._agglomerans_UAEU18	P._agglomerans_ANP8	P._agglomerans_4188	P._agglomerans_P5	P._agglomerans_Tx10	P._agglomerans_L15	P._agglomerans_P10c	P._agglomerans_Eh318	P._agglomerans_824−1	P._agglomerans_E325	P._agglomerans_C1	P._agglomerans_4	P._agglomerans_R190
P._agglomerans_LMAE−2	100	99	99	99	99	99	99	99	97	97	97	97	97	97	97	97	97	97
P._agglomerans_DAPP−PG734	99	100	99	99	99	99	99	99	97	98	98	98	98	97	98	98	97	97
P._agglomerans_KM1	99	99	100	99	99	99	99	99	97	97	97	97	97	97	97	97	97	97
P._agglomerans_BD_1274	99	99	99	100	99	99	99	99	97	97	97	97	98	97	97	97	97	97
P._agglomerans_Pa	99	99	99	99	100	99	99	99	97	98	97	97	98	97	98	97	97	97
P._agglomerans_UAEU18	99	99	99	99	99	100	99	99	97	97	97	97	97	97	97	97	97	97
P._agglomerans_ANP8	99	99	99	99	99	99	100	99	97	97	97	97	98	97	98	97	97	97
P._agglomerans_4188	99	99	99	99	99	99	99	100	97	98	97	97	97	97	97	98	97	97
P._agglomerans_P5	97	97	97	97	97	97	97	97	100	97	97	97	97	97	97	97	97	97
P._agglomerans_Tx10	97	98	97	97	98	97	97	98	97	100	99	99	99	99	99	99	99	99
P._agglomerans_L15	97	98	97	97	97	97	97	97	97	99	100	99	99	99	99	99	99	99
P._agglomerans_P10c	97	98	97	97	97	97	97	97	97	99	99	100	99	99	99	99	99	99
P._agglomerans_Eh318	97	98	97	98	98	97	98	97	97	99	99	99	100	99	99	99	99	99
P._agglomerans_824−1	97	97	97	97	97	97	97	97	97	99	99	99	99	100	99	98	99	98
P._agglomerans_E325	97	98	97	97	98	97	98	97	97	99	99	99	99	99	100	99	99	99
P._agglomerans_C1	97	98	97	97	97	97	97	98	97	99	99	99	99	98	99	100	99	99
P._agglomerans_4	97	97	97	97	97	97	97	97	97	99	99	99	99	99	99	99	100	99
P._agglomerans_R190	97	97	97	97	97	97	97	97	97	99	99	99	99	98	99	99	99	100
